# PGE2 inhibits neutrophil phagocytosis through the EP2R–cAMP–PTEN pathway

**DOI:** 10.1002/iid3.662

**Published:** 2022-06-10

**Authors:** Zixuan Wang, Xinyuan Wei, Caili Ji, Wenhua Yu, Chuanwang Song, Caizhi Wang

**Affiliations:** ^1^ Department of Immunology, School of Laboratory Medicine Bengbu Medical College Bengbu Anhui P.R. China; ^2^ Anhui Province Key Laboratory of Immunology in Chronic Diseases, Bengbu Medical College Bengbu Anhui P.R. China; ^3^ Department of Obstetrics and Gynecology the First Affiliated Hospital of Bengbu Medical College Bengbu China Anhui

**Keywords:** neutrophil, phagocytosis, prostaglandin E2 (PGE2), EP2 receptor, cAMP, phosphatase and tensin homolog (PTEN)

## Abstract

Prostaglandin E2 (PGE2) is a potent lipid mediator of inflammation that modulates immune cell function by binding to unique G protein‐coupled receptors (EP receptors). PGE2 production increases during microbial infection and inflammation. In this study, we assessed the effect of PGE2 on the phagocytosis of bacteria by neutrophils, which are key players during infection and inflammation. We also looked for specific EP receptor signaling pathways that contributed to the neutrophil phagocytic activity. PGE2 (50–1000 ng/ml) inhibited the phagocytosis of *Escherichia coli* by HL‐60 human neutrophils in a concentration‐dependent manner. Inhibition of neutrophil phagocytosis by PGE2 correlated with increased intracellular cyclic adenosine monophosphate (cAMP) production, and forskolin, an adenosyl cyclase agonist, confirmed the inhibitory effect of cAMP stimulation on neutrophil phagocytosis. The expression of EP2 receptors by HL‐60 cells was confirmed by western blot analysis, and selective agonism of EP2 receptors mimicked the inhibition of phagocytosis by PGE2. The EP2 receptor antagonist AH‐6089 partially blocked the inhibition of neutrophil phagocytosis PGE2. Specific inhibition of phosphatase and tensin homolog (PTEN) enzyme attenuated the inhibition of neutrophil phagocytosis by PGE2, and both PGE2 and increased intracellular cAMP increased neutrophil PTEN activity, which was associated with decreased PTEN phosphorylation. The results support negative regulation of the antimicrobial activity of neutrophils (i.e., phagocytosis), which has important implications for the future management of bacterial infections.

## Graphical abstract

In this study, we assessed the effect of prostaglandin E2 (PGE2) on the phagocytosis of bacteria by neutrophils, which are key players during infection and inflammation. Specific inhibition of phosphatase and tensin homolog (PTEN) enzyme attenuated the inhibition of neutrophil phagocytosis by PGE2, and both PGE2 and increased intracellular cyclic adenosine monophosphate (cAMP) increased neutrophil PTEN activity, which was associated with decreased PTEN phosphorylation. The results support negative regulation of the antimicrobial activity of neutrophils (i.e., phagocytosis), which has important implications for the future management of bacterial infections.

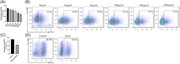

## 1 INTRODUCTION

Neutrophils are the most abundant circulating leukocytes in humans. They are terminally differentiated cells that are constantly renewed by production in the bone marrow. They are effector cells of the innate immune system and are active in inflammation.^1^, ^2^ Neutrophils have antimicrobial activity mediated by reactive oxygen intermediates and hydrolases capable of clearing invading pathogens, which places them in the first line of defense against bacteria and fungi.^3^ Phagocytosis of invading pathogens by neutrophils is an efficient defense against infectious diseases, involves particles that are larger than 0.5 µM in diameter, and is mainly performed by professional phagocytes such as neutrophils and macrophages.^4^ The antimicrobial activity of innate immunity is modulated by autocrine and paracrine secretion of chemokines, cytokines, and phospholipids.^5^, ^6^ Prostaglandins are lipid metabolites of arachidonic acid that are potent mediators of innate immunity, and PGE2 has been shown to regulate immune and inflammatory responses.^7^ It is a metabolite of the cyclooxygenase (COX) cascade, which includes two isoforms, COX‐1 and COX‐2, and PGE synthase. Inflammation is accompanied by increased PGE2 production, and PGE2 has both proinflammatory and anti‐inflammatory properties. PGE2 production increases if an infection occurs, and overproduction has been reported to increase susceptibility to infection with aging and in the presence of cancer, malnutrition, and other conditions.^8^, ^9^ The net effect of PGE2 is inhibitory in the context of infection, which is supported by studies demonstrating that COX inhibitors increase the survival and clearance of microorganisms in animal models of infection.^10^, ^11^ PGE2 was shown to inhibit phagocytosis of bacteria by monocytes or macrophages,^12^ but it is not known whether PGE2 has an effect on polymorphonuclear (PMN) phagocytosis of bacteria. This study showed that PGE2 inhibited PMN phagocytosis of *Escherichia Coli* (*E. coli*) via the E‐series of prostaglandin receptors type 2 (EP2R)–cAMP–phosphatase and tensin homolog (PTEN) pathway.

## 2 MATERIALS AND METHODS

### 2.1 HL‐60 cell culture

HL‐60 human neutrophil cells were purchased from Wuhan Proceeds Company. Cells were seeded in Iscove's modified Dulbecco's medium (Hyclone) containing 20% fetal bovine serum (FBS) and cultured at 37°C with 5% CO_2_. Cells were passaged every 2–3 days.

### 2.2 Isolation and culture of neutrophils

Peripheral venous blood was drawn from healthy adults, and erythrocytes were sedimented with 3% Dextran T‐500 solution. The supernatant was removed and Ficoll solution was used to separate the mononuclear cells. The granulocyte layer was removed and 0.83% NH_4_Cl solution was added to lyse the red blood cells. The neutrophils were obtained after washing, resuspended in Roswell Park Memorial Institute (RPMI)1640 medium (Gibco) containing 10% FBS, 1 × 10^5^ cells were added to each well of a 12‐well plate, and cultured at 37°C and 5% CO_2_.

### 2.3 Phagocytosis assay

HL‐60 cells were cultured with fluorescein isothiocyanate (FITC)‐labeled *E. coli* at a ratio of 1:100 for 2 h. Primary neutrophils were cultured with FITC‐labeled *E. coli* at a ratio of 1:10 for 15 min. Extracellular fluorescence was quenched with trypan blue, the cells were washed twice with PBS and fixed in 300 µl of 1% paraformaldehyde. Phagocytosis of fluorescently labeled *E. coli* was assayed by fluorescence‐activated cell sorting with a DxP Athena™ flow cytometer (Cytek Biosciences) and the results were reported as the percentage of the total cell sample that had phagocytized *E. coli*.

### 2.4 **Enzyme‐linked immunoassay** (ELISA) of intracellular cAMP

HL‐60 cells were seeded in six‐well plates at a density of 1 × 10^6^ cells/well. The cells were collected after treatment, lysed by repeated freezing and thawing, and the cell lysate was used in the ELISA procedures following the cAMP kit (Mlbio) manufacturers' instructions. Optical density was measured at 450 nm in both assays, and the intracellular cAMP concentrations were calculated using standard curves.

### 2.5 PTEN activity assay

HL‐60 cells were seeded in 6‐well plates at a density of 1 × 10^6^ cells/well, collected after treatment, lysed, and the protein concentration of the lysates was determined with a bicinchoninic acid assay (Beyotime Biotechnology). In the colorimetric PTEN activity assay kits that were used (Genmed Scientific), the free phosphate released by the dephosphorylation of PTEN reacted with malachite green dye, and the optical density was measured at 630 nm. PTEN activity was reflected by the phosphorus concentration in each group of samples measured using a standard curve.

### 2.6 Immunoblotting

Cells in each group were lysed with NP‐40 protein lysis buffer (Beyotime Biotechnology) containing the protease inhibitor phenylmethylsulfonyl fluoride for 30 min on ice. A phosphatase inhibitor was added when performing assays of phosphorylated protein. Protein concentration was determined with a bicinchoninic acid assay. The extracted proteins were separated by 10% sodium dodecyl sulfate–polyacrylamide gel electrophoresis and transferred to polyvinylidene difluoride membranes (Millipore). Membranes were blocked with 5% nonfat milk solution for 2 h at room temperature and incubated overnight at 4°C with specific primary antibodies, which included anti‐PTGER2 prostaglandin E receptor antibody (1:2000; Affinity), anti‐PTEN (1:5000; Abcam), and anti‐phosphotyrosine (1:1000; PY20; Abcam). The membranes were washed and incubated with horseradish peroxidase (HRP)‐conjugated goat anti‐rabbit IgG or HRP‐conjugated goat anti‐mouse IgG (1:5000; Beyotime Biotechnology) for 1.5 h at room temperature. The protein bands were visualized with an enhanced chemiluminescence kit (Tanon).

### 2.7 Statistical analysis

The experimental data were expressed as mean ± standard deviation. SPSS 16.0 software was used for data analysis. One‐way analysis of variance (ANOVA) was used for comparison between multiple groups, and *t* test was used for comparison between two groups. *p* < .05 was considered statistically significant.

## 3 RESULTS

### 3.1 PGE2 prevents phagocytosis of neutrophils

**Figure 1:**
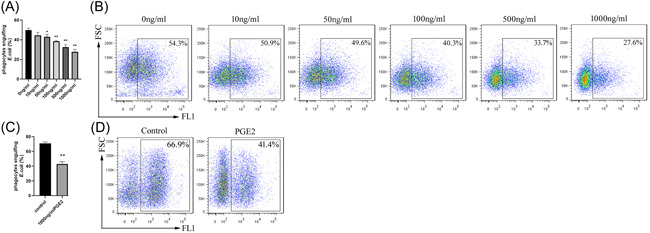
PGE2 prevents phagocytosis of neutrophils. HL‐60 neutrophil cells were seeded in 12‐well plates at 1 × 10^5^ cells/well with 0, 10, 50, 100, 500, and 1000 ng/ml PGE2 to stimulate HL‐60 cells for 12 h. FITC‐labelled *Escherichia coli* were added at a ratio of 1:100 and incubated for 2 h. Following trypan blue quenching of extracellular fluorescence, (A) the percentage of phagocytotic HL‐60 cells was determined by flow cytometry. (B) A representative flow cytometry plot. *p < .05, **p < .01, compared with 0 ng/ml. PGE2 (1 μg/ml) was used to stimulate neutrophils freshly isolated from human venous peripheral blood for 2 h and FITC‐*E. coli* was added at a ratio of 1:10 for 15 min. After trypan blue quenching of extracellular fluorescence, (C) the percentage of neutrophil phagocytosis was assayed by flow cytometry. (D) A representative flow cytometry plot. **p < .01 compared with the unstimulated control group. PGE2, prostaglandin E2.

Exogenous PGE2 has been reported to prevent phagocytosis of macrophages,^13^ and in our experimental system, starting with a concentration of 50 ng/ml, PGE2 significantly inhibited the phagocytosis of *E. coli* by HL‐60 cells. At 1000 ng/ml, PGE2 reduced the phagocytic percentage of HL‐60 cells by about 50% (Figure 1A,B). PGE2 also inhibited the phagocytosis of *E. coli* by freshly isolated neutrophils (Figure 1C,D).

### 3.2 PGE2 leads to increased cAMP production in neutrophils through EP2 receptors

**Figure 2:**
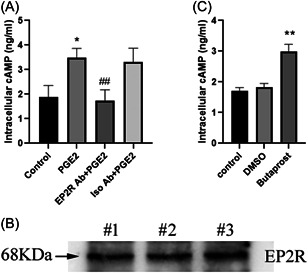
PGE2 binding to EP2 receptors increases cAMP production in neutrophils. (A) HL‐60 cells were pretreated with EP2R blocking antibody (1 μg/ml) or isotype control antibody (1 μg/ml) for 6 h, and then stimulated with PGE2 (1 μg/ml) for 12 h. The cells were collected and the cAMP content was determined by ELISA. *p < .05 compared with the control group. ^##^ *p* < .01 compared with the PGE2 group using the results of three samples of HL‐60 cells. (B) The expression of EP2R in HL‐60 cells was determined by western blot analysis. (C) HL‐60 cells were treated with 1 μg/ml butaprost for 12 h, lysed to measure intracellular cAMP concentration. **p < .01 compared with the control group. ELISA, enzyme‐linked immunoassay; cAMP, cyclic adenosine monophosphate; EP2R, E‐series of prostaglandin receptors type 2; PGE2, prostaglandin E2.

The biological activity of PGE2 is mediated by binding by a G protein‐coupled EP2 receptor on the cell membrane. The EP2 receptor activates adenylate cyclase, which catalyzes the production of cAMP.^14^ We measured intracellular cAMP levels in HL‐60 cells in response to PGE2. Stimulation of PGE2 increased cAMP production in HL‐60 cells (Figure 2A), suggesting that EP2 receptors were involved. Immunoblotting assays showed that HL‐60 cells expressed the EP2 receptor (Figure 2B), and a PGE2 receptor‐blocking antibody blocked the upregulation of cAMP production in HL‐60 cells (Figure 2A), indicating that EP2 receptor‐mediated PGE2‐induced cAMP production. EP2 receptor agonists caused an increase in cAMP production in HL‐60 cells (Figure 2C). The results suggest that PGE2 binding to the EP2 receptor led to increased cAMP production in neutrophils.

### 3.3 EP2 receptor agonists prevent phagocytosis of neutrophils

**Figure 3:**
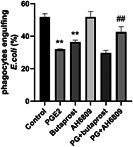
EP2 receptor agonists prevent phagocytosis of neutrophils. HL‐60 cells were stimulated with 1 μg/ml butaprost or 5 μg/ml AH‐6809 for 12 h in the presence or absence of 1 μg/ml PGE2 before incubation with FITC‐*Escherichia coli* (1:100) for 2 h. The percentage of *E. coli* phagocytosed by HL‐60 cells was determined by flow cytometry. **p < .01 compared with the control group; ^##^ *p* < .01 compared with the PGE2 group. PGE2, prostaglandin E2.

To investigate the role of neutrophil EP2 receptors in the inhibition of phagocytosis by PGE2, we treated HL‐60 cells with the EP2 receptor agonist butaprost or the EP2 receptor antagonist AH‐6809 in the presence or absence of PGE2. The effects on neutrophils are shown in Figure 3. At a concentration of 1 μg/ml, butaprost inhibition of neutrophil phagocytosis was similar to that of 1 μg/ml PGE2. The AH‐6809 EP2 receptor antagonist partially reversed the inhibition of neutrophil phagocytosis by PGE2, showing that PGE2 binding to its EP2 receptor inhibited neutrophil phagocytosis.

### 3.4 Elevation of cAMP inhibits neutrophil phagocytosis

**Figure 4:**
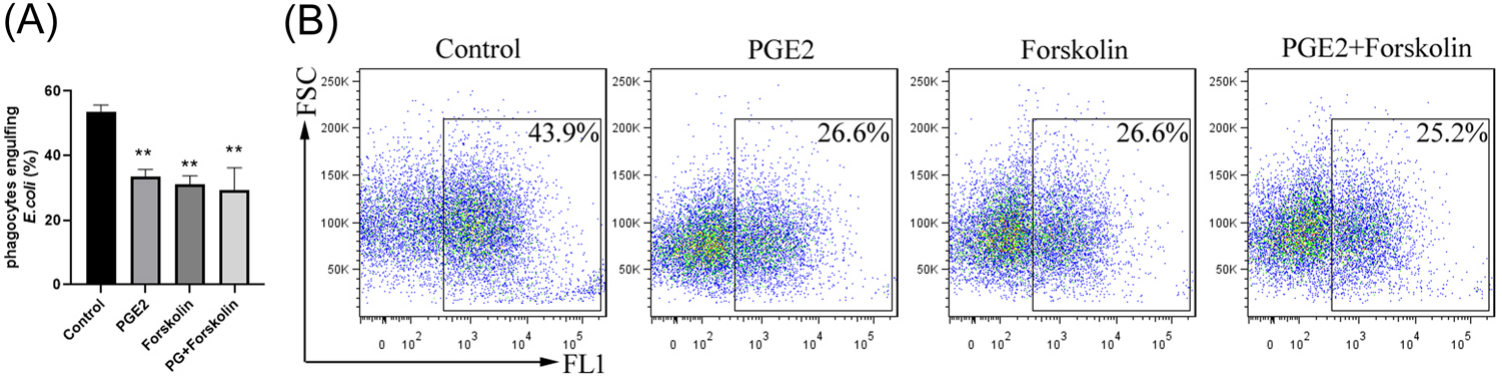
cAMP elevation inhibits neutrophil phagocytosis. HL‐60 cells were stimulated with 1 μg/ml PGE2 and 5 μg/ml either alone or combined for 12 h before FITC‐*Escherichia coli* were (1:100) were added. After 2 h, the percentage of phagocytes HL‐60 cells was determined by flow cytometry. (A) Percentage of phagocytic cells, and (B) a representative flow cytometry graph, **p < .01 compared with the control group. cAMP, cyclic adenosine monophosphate; PGE2, prostaglandin E2.

Our results are consistent with the findings of others that an increase of intracellular cAMP is associated with the inhibition of phagocytosis.^15^ We investigated the causal relationship between PGE2‐induced cAMP production and inhibition of neutrophil phagocytosis. Forskolin is a direct agonist of adenosyl cyclase in neutrophils, and at 5 μg/ml it inhibited phagocytosis in HL‐60 cells to approximately the same extent as 1 μg/ml PGE2 (Figure 4A,B). The results indicate that inhibition of neutrophil phagocytosis was associated with increased production of intracellular cAMP.

### 3.5 PGE2 inhibits neutrophil phagocytosis in a PTEN‐dependent manner

**Figure 5:**
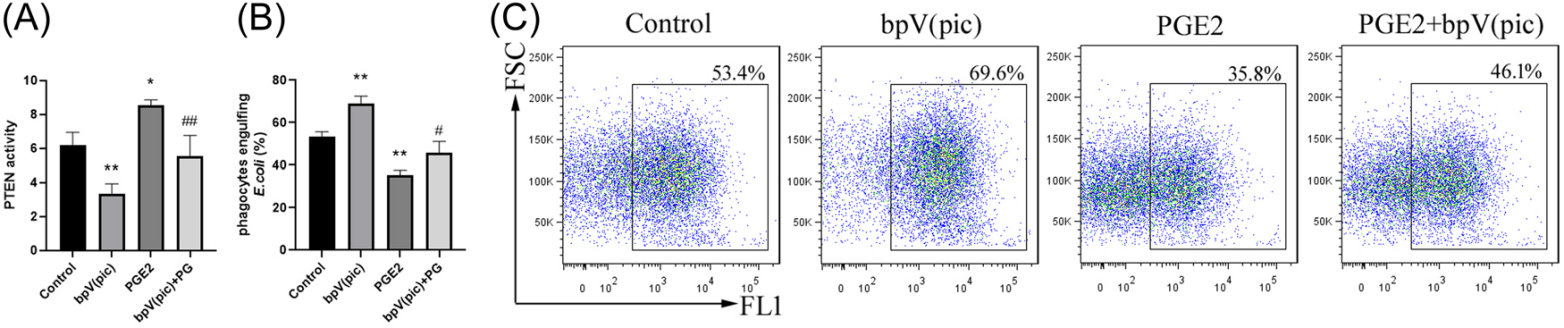
PGE2 inhibits neutrophil phagocytosis in a PTEN‐dependent manner. HL‐60 cells were pretreated with 500 ng/ml bpV(pic), a PTEN inhibitor, for 2 h, stimulated with 1 μg/ml PGE2 for 12 h, and then incubated with FITC‐*Escherichia coli* (1:100) for 2 h. (A) PTEN activity was assayed by phosphorus colorimetry. *p < .05, **p < .01 compared with the control group, ^##^ *p* < .01 compared with the PGE2 group. The percentage of phagocytic cells in each group (B) was determined by flow cytometry. (C) A representative flow cytometry plot. **p < .01 compared with the control group; ^#^ *p* < .05 compared with the PGE2 group. PGE2, prostaglandin E2; PTEN, phosphatase and tensin homolog.

PIP3 is required for phagocytic cup formation. PTEN is a phosphatase that dephosphorylates PIP3, thereby preventing phagocytosis.^16^ We wondered whether PGE2 inhibition of neutrophil phagocytosis was associated with PTEN activity. We found that PTEN activity in HL‐60 cells was increased after PGE2 stimulation, but the increase was significantly attenuated by the PTEN‐specific inhibitor bpV(pic) (Figure 5A). When HL‐60 cells were pretreated with bpV(pic) and then challenged with FITC‐*E. coli*, PGE2‐mediated inhibition of phagocytosis was reduced (Figure 5B,C). The results suggest that the inhibitory effect of PGE2 on neutrophil phagocytosis of *E. coli* was dependent on PTEN activity.

### 3.6 PGE2 and cAMP lead to dephosphorylation of PTEN and increase PTEN activity

**Figure 6:**
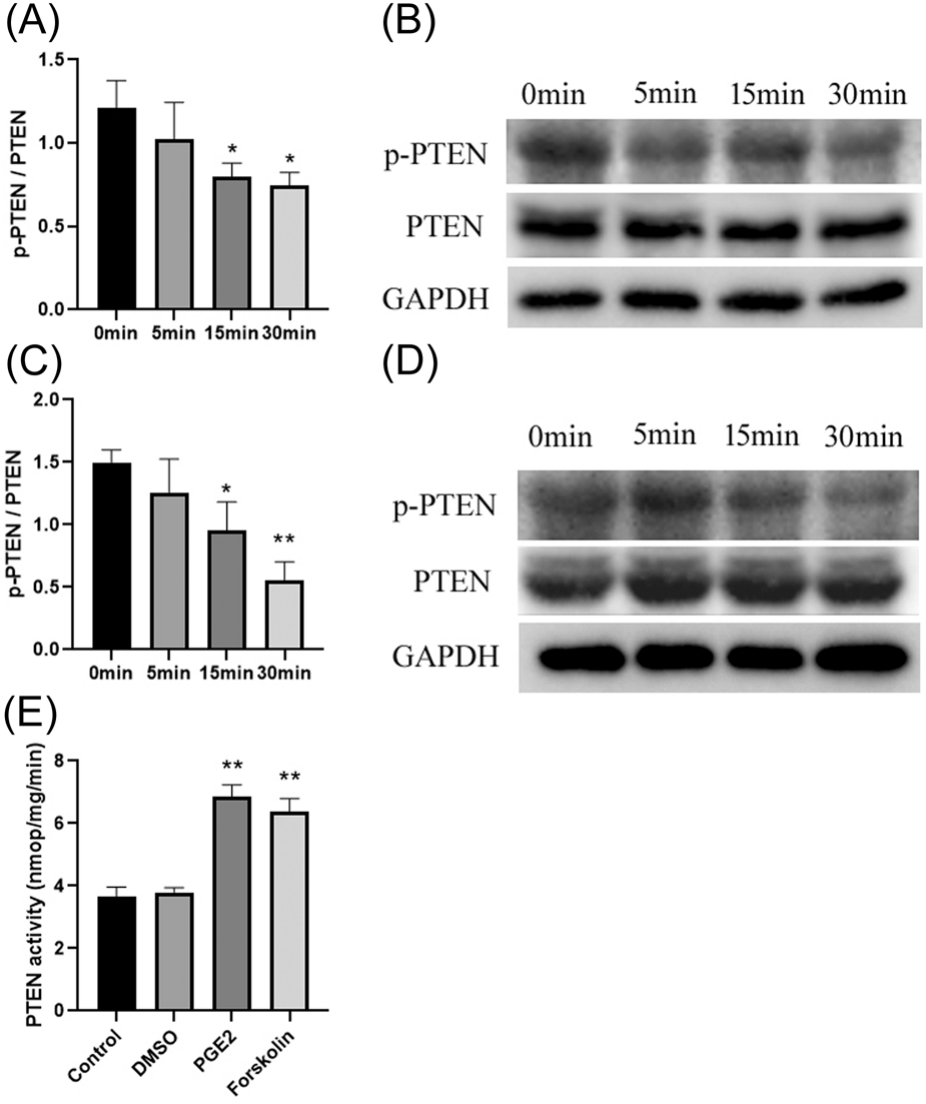
PGE2 and cAMP lead to dephosphorylation of PTEN and increase PTEN activity. HL‐60 cells were stimulated with 1 μg/ml PGE2 (A, B) or 5 μg/ml forskolin (C, D) for 5, 15, or 30 min. Total PTEN and phosphorylated PTEN were assayed by western blot analysis to determine (A and C) relative protein expression. (B and D) Representative immunoblots. *p < .05, **p < .01 compared with 0 min. (E) After HL‐60 cells were treated with 1 μg/ml PGE2 or 5 μg/ml forskolin for 30 min, PTEN activity was determined. **p < .01 compared with the control group. cAMP, cyclic adenosine monophosphate; PGE2, prostaglandin E2; PTEN, phosphatase and tensin homolog.

PTEN phosphorylation is negatively correlated with PTEN activity. Baseline PTEN phosphorylation was assayed in untreated neutrophils, and treatment with PGE2 resulted in PTEN dephosphorylation (Figure 6A,B). The increase of cAMP induced by the adenosyl cyclase agonist forskolin, also resulted in significant PTEN dephosphorylation (Figure 6C,D). As expected, stimulation of PGE2 and forskolin increased PTEN enzyme activity in neutrophils (Figure 6E). The data show that PGE2 increase neutrophil PTEN activity by promotion of PTEN dephosphorylation, and PGE2‐induced increase in cAMP production.

## 4 DISCUSSION

In this study, PGE2 inhibited the phagocytosis of *E. coli* by neutrophils. PGE2, a lipid mediator derived from the fatty acid arachidonic acid, is the most widely studied member of the PG family of inflammatory factors. Although PGE2 has a short half‐life, it has a key role in mediating inflammation and a variety of biological processes in vivo. Previous studies have demonstrated that PGE2 regulates the activation, maturation, differentiation, migration, and other activities of immune cells.^7^, ^17^ For example, PGE2 reduces the production of interleukin (IL)‐12 in monocytes or dendritic, which selectively prevents the differentiation of naïve CD4^+^ T cells into Th1 cells.^18^, ^19^ Chen et al. showed that PGE2 suppressed the function of antigen‐specific CD8^+^T cells and promoted the apoptosis of CD8^+^T cells.^20^ PGE2 greatly reduced the killing of bacteria by macrophages by inhibiting the production of H_2_O_2_.^21^ PGE2 also regulates the phagocytosis of cells. He et al. showed that PGE2 inhibited the phagocytosis of microglia.^22^ PGE2 has also been reported to inhibit the phagocytosis of bacteria by alveolar macrophages. Our results suggest that PGE2 prevents phagocytosis of *E. coli* by neutrophils.

PGE2 functions through binding to four transmembrane G protein‐coupled prostaglandin E receptors, EP1–4 receptors. The human EP2 receptor consists of 358 amino acids, and the receptor is coupled to the alpha subunit of the Gs protein. Binding leads to an increase in intracellular cAMP. EP2 receptors participate in most of the immunomodulatory effects of PGE2 in innate and adaptive immunity.^14^ For example, PGE2 prevents the activity of natural killer (NK) cells through the EP2 receptor.^23^ PGE2 also inhibits the proliferation of neuronal cells through the EP2 receptor, and the cAMP signaling pathway is involved in PGE2‐induced neuronal differentiation.^24^ Our findings show that PGE2 led to inhibition of neutrophil phagocytosis through the EP2 receptor and that induction of cAMP signaling was involved. Lu et al. recently showed that EP2 and EP4 receptors are coupled with activation of the cAMP pathway.^25^ Therefore, it may be a very important field to explore the role of EP4 receptor in PGE2 preventing neutrophils from phagocytosing bacteria and its relationship with EP2 receptor.

cAMP is a versatile cellular second messenger that regulates cell activation, survival, proliferation, migration, and other activities.^26^ cAMP production is triggered by activation of membrane receptors, most of which are G protein‐coupled receptors that activate intracellular adenylate cyclase and conversion of ATP to cAMP.^27^ Elevation of intracellular cAMP regulates the effector functions of various innate immune cells including monocytes, macrophages, and neutrophils. Elevation of cAMP also activates Notch signaling in monocytes and increases the expression of transducin‐like enhancer, which may be a mechanism by which cAMP suppresses immune responses.^28^ cAMP promotes the expression of cytokines such as interleukin‐10 (IL‐10) and IL‐13 and markers such as CD206 and Arg1 that occurs during the transformation of macrophages to the M2 type.^29^ cAMP signaling has also been reported to enhance neutrophil adhesion and chemotaxis, induce actin polymerization by activating protein kinase A, and inhibit the phagocytosis of bacteria by macrophages.^30^, ^31^ Zalavary et al. reported that elevated cAMP correlated with inhibition of neutrophil phagocytosis,^32^ which is consistent with our findings.

In this study, increased cAMP enhanced PTEN activity and inhibited neutrophil phagocytosis. PTEN was first identified as a tumor suppressor gene in 1997, and subsequently was found to be negative regulator of cell growth and proliferation. It is one of the most frequently mutated genes in tumors. The PTEN gene encodes a ubiquitously expressed dual‐specificity phosphatase that acts as an important regulator of cellular signaling and immune cell function.^33^, ^34^ For example, the overexpression of PTEN blocks the cytolytic activity of NK cells, and loss of PTEN increases the killing function of NK cells.^35^ Myeloid PTEN deletion increases neutrophil chemotaxis, superoxide production, and alters dendritic cell function, resulting in impaired CD8^+^ T‐cell activation.^36^, ^37^ PTEN deficiency in macrophages results in increased chemotaxis production of proinflammatory cytokines and increased production of anti‐inflammatory cytokines.^38^ The classical role of PTEN is as a negative regulator of PI3K/Akt signaling through its lipid phosphatase activity, and it directly antagonizes the action of PI3K, which is an essential signaling component involved in phagocytosis.^39^ The role of PTEN as a negative regulator of PI3K and its effect on phagocytosis has attracted attention. In vitro silencing of PTEN promotes endocytosis of low‐density lipoprotein in mouse podocytes, and PTEN overexpression inhibits the endocytosis of lipoprotein in podocytes. Downregulation of PTEN in podocytes may thus contribute to the pathogenesis of obesity‐related glomerulopathy.^40^ PTEN directly activates actin depolymerization factor cofilin‐1 in macrophages to inhibit the phagocytosis of *Candida albicans*.^13^ Consistent with those findings, our results show that PTEN activity also inhibits neutrophil phagocytosis.

Overall, the study results demonstrated that PGE2 inhibited the phagocytosis of *E. coli* by neutrophils, and that this immunosuppressive activity was mediated by activation of PTEN following binding to its transmembrane EP2 receptor and the resulting increase in cAMP. The results have important implications for future efforts to prevent and manage bacterial infections, especially in immunosuppressed individuals who may produce excess PGE2.

## AUTHOR CONTRIBUTIONS

Chuanwang Song and Caizhi Wang conceived and designed the experiments. Zixuan Wang, Xinyuan Wei, Caili Ji, and Wenhua Yu performed the experiments. Chuanwang Song and Caizhi Wang analyzed the data and wrote the manuscript.

## CONFLICT OF INTEREST

The authors declare no conflict of interest.
